# A high gain multiband offset MIMO antenna based on a planar log-periodic array for Ku/K-band applications

**DOI:** 10.1038/s41598-022-07866-1

**Published:** 2022-03-08

**Authors:** Mohammad M. Fakharian, Mohammad Alibakhshikenari, Chan Hwang See, Raed Abd-Alhameed

**Affiliations:** 1 Faculty of Engineering, University of Garmsar, Garmsar, Iran; 2 Department of Signal Theory and Communications, Universidad Carlos III de Madrid, 28911 Leganés, Madrid, Spain; 3 School of Engineering and the Built Environment, Edinburgh Napier University, Edinburgh, EH10 5DT UK; 4 Faculty of Engineering and Informatics, University of Bradford, Bradford, BD7 1DP UK

**Keywords:** Electrical and electronic engineering, Applied physics

## Abstract

An offset quad-element, two-port, high-gain, and multiband multiple-input multiple-output (MIMO) planar antenna based on a log-periodic dipole array (LPDA) for Ku/K-band wireless communications is proposed, in this paper. A single element antenna has been designed starting from Carrel’s theory and then optimized with a 50-Ω microstrip feed-line with two orthogonal branches that results mainly in a broadside radiation pattern and improves diversity parameters. For experimental confirmation, the designed structure is printed on an RT-5880 substrate with a thickness of 1.57 mm. The total substrate dimensions of the MIMO antenna are 55 × 45 mm^2^. According to the measured results, the designed structure is capable of working at 1.3% (12.82–12.98 GHz), 3.1% (13.54–13.96 GHz), 2.3% (14.81–15.15 GHz), 4.5% (17.7–18.52 GHz), and 4.6% (21.1–22.1 GHz) frequency bands. Additionally, the proposed MIMO antenna attains a peak gain of 4.2–10.7 dBi with maximum element isolation of 23.5 dB, without the use of any decoupling structure. Furthermore, the analysis of MIMO performance metrics such as the envelope correlation coefficient (ECC) and mean effective gain (MEG) validates good characteristics, and field correlation performance over the operating band. The proposed design is an appropriate option for multiband MIMO applications for various wireless systems in Ku/K-bands.

## Introduction

Recently, because of the rapid developments in wireless systems, multiple-input multiple-output (MIMO) technologies have attracted much attention from engineering institutes and the research community. It is believed that MIMO systems will be crucial to improve the performance of wireless communications^1^. They can minimize the effect of multipath fading and channel interference, enhance the spectral efficiency, improve the channel capacity, create a high data rate, etc.^2^. Therefore, combining multiple band antennas with MIMO systems has become essential.

In the past few years, several multiband MIMO antenna technologies have been proposed in the literature, that can provide compatibility with existing wireless systems and cover multiple frequencies^3–9^. To design the MIMO antenna, we must consider not only the compact structure but also the reduction of the mutual coupling among adjacent elements required. The higher isolation among the MIMO antenna elements would affect the system throughput^10^. Therefore, many techniques have been investigated in the literature to obtain high mutual coupling among the elements of MIMO antennas. These decoupling approaches include frequency selective surfaces for displacing current among elements^11^, hybrid feeding with orthogonal modes^12–15^, parasitic structures at the expense of space and size^16^, metasurface shielding^16,17^, neutralization lines^18,19^, artificial metamaterials^20–23^, etc.^24,25^. However, some of these methods, such as parasitic structures and neutralization lines, may deteriorate the impedance matching of the MIMO antenna, therefore making it challenging to obtain a multiband operation simultaneously with low correlation and high isolation of ports. Additionally, designing the decoupling structures for the MIMO system needs extra considerable effort and extra space in the MIMO antenna implementation. Among these methods, the hybrid feeding with orthogonal mode can be used to fine-tune the level of mutual coupling reduction and the matching of antennas without any decoupling structure.

On the other hand, in wireless communications, atmosphere and rain are a great challenge because higher frequencies become unfeasible. In^26^, the atmospheric and rain attenuation experienced by different frequency bands ranging from 0 to 400 GHz are discussed. Additionally, the utilization of high frequencies such as Ku/K bands, raises other challenges, such as free space path loss, adjacent channel interference, and multipath fading. The loss of path can be compensated by high gain antennas, whereas the MIMO antennas are crucial to minimize the effect of multipath fading and channel interference, as mentioned. Therefore, a high gain antenna with a MIMO structure and multiband characteristics can resolve these issues and improve the channel capacity and spectrum efficiency with no need to increase the input power in the Ku/K bands. The main applications of Ku/K bands are radar, synthetic aperture radar (SAR), surface movement radar (SMR), air surface detection equipment (ASDE), air traffic management (ATM), high-performance aircraft, spacecraft, satellite communications systems, direct broadcast satellite (DBS) services, and other modern communications systems which require short range and very high resolution.

This article proposes an integrated 4-element offset MIMO antenna system based on a planar log-periodic array (PLPA) to improve the multiband, radiation gain, and efficiency characteristics. PLPAs are multiband, low-profile, and non-frequency- dependent antennas, that are practically built out of resonant elements^8,27–32^. Among the various designed structures in PLPAs, planar dipole arrays have the disadvantage of complex feeding, while, other PLPAs can be appropriately fed by a microstrip line, which simplifies the structure of feeding and reduces cost. To date, many designs of microstrip-fed PLPAs with different feeding structures have been proposed. In patch radiators, the design methodology for the PLPA is similar to that presented by Carrel for log-periodic dipole arrays (LPDAs)^33^, since the resonant elements are dipoles. Therefore, in this work, the design method has been developed, considering this resonant nature. Furthermore, to enhance antenna design and diversity parameters, the designed MIMO radiator with diverse branches has been proposed. The proposed MIMO antenna can operate at a much higher frequency for potential Ku/K-band applications, which, to the best of the authors’ knowledge, is presented for the first time. The offsetting of ports is applied in the design to better use the diagonal space of the implementation and orthogonally assemble antenna elements in the anti-parallel mode to reduce the mutual coupling among the MIMO antenna elements^34,35^. According to^36^, a common ground plane is used in the proposed MIMO antenna system to obtain the same voltage in the antenna elements and a stable operation. In the following sections, the detailed design procedure and results achieved using the 3-D electromagnetic simulator CST Microwave Studio and experimental evaluation are presented and discussed. MIMO performance metrics are also calculated and discussed.

## Configuration of the proposed antenna and design

**Figure 1:**
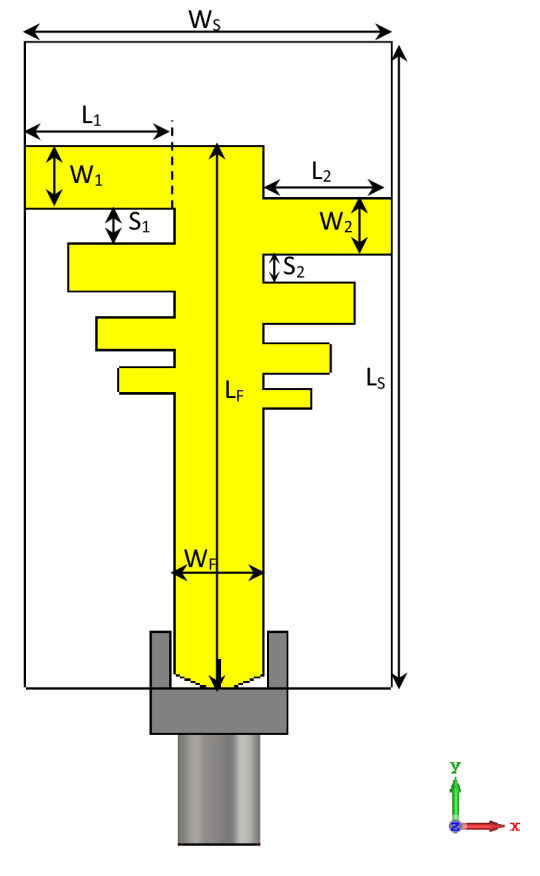
Geometry of the unit PLPA element.

**Figure 2:**
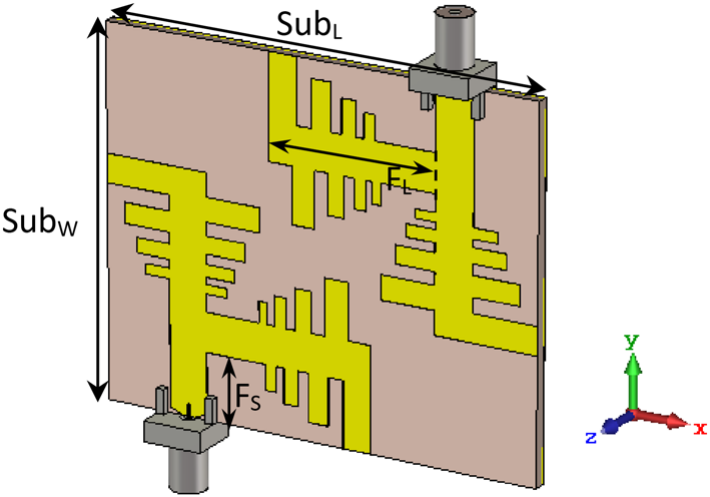
Geometric layout of the proposed multiband MIMO antenna.

The proposed four-element MIMO antenna is fabricated on an RT-5880 substrate with a thickness of 1.57 mm, loss tangent of 0.0009, and permittivity of 2.2. Figures 1 and 2 show the geometrical layout of the unit element and MIMO configuration, respectively, while the modified parametric values for the design parameters are listed in Table 1. The total size of the two-port MIMO prototype is only 55 × 45 mm^2^, while the size of a single antenna is approximately 35 × 20 mm^2^ (~ 1.4λ_0_ × 0.8λ_0_, where λ_0_ is the wavelength of the free-space centered at 12 GHz). A finite ground plane made of copper is used to back the substrate. Copper with a very stable conductivity of 5.8 × 10^7^ S/m is applied to the radiating elements, because its effect on impedance matching is shallow. The MIMO antenna is designed based on PLPA and two 50-Ω ports on offset positions to utilize the diagonal space. A common microstrip feed configuration with two orthogonal branches is utilized to construct a 2 × 2 MIMO antenna with suitable spacing between the branches and to obtain mostly broadside or dual-beam radiations. The effect of diversity performance has also been achieved to cover the signals in horizontal and vertical directions to maintain the reliability of receiving/transmission and to minimize loss of signal. The design progress is first revealed by describing single-element antennas and then followed by the MIMO configuration.

**Table 1:** Modified parametric values of the proposed multiband antenna.

Parameter	Value (mm)	Parameter	Value (mm)
Sub_L_	55	W_F_	4.8
Sub_W_	45	L_1_	8.1
F_L_	21.1	W_1_	3.5
F_S_	8.2	S_1_	1.9
L_S_	35	L_2_	6.9
W_S_	20	W_2_	3
L_F_	29	S_2_	1.6

### Single antenna design

The structure of the unit PLPA element, as shown in Fig. 1, serves as a building block for the two ports of the quad element MIMO antenna. The antenna is provided with a 50-Ω matched microstrip feed-line, in which the width (W_f_ = 4.8 mm) of the feed-line is tuned according to the characteristic equations of the microstrip transmission lines. An entire ground plane is printed on the back of the antenna, to obtain better performance by reducing the antenna radiated wave flow in the back direction and to obtain a maximum gain, which is essential in Ku/K band applications.

The PLPA is made of a microstrip transmission line loaded with several log-periodically-scaled radiating elements in the logarithmic function of frequency represented by log-periodic distances in an alternate way^30^. PLPA antennas are typically characterized by passive regions and active regions. The active region of the antenna is near the radiating elements whose lengths are approximately half the wavelength, and this region is shifted inside the array from longer to shorter elements as the frequency increases. The shorter and longer elements than half the wavelength are included in the passive region and do not radiate properly.

The number of elements, their dimensions, and the spacing between them should be carefully chosen to obtain the proper multiband performance for the desired frequency range in the PLPA antenna. In the PLPA antenna, there are *n* elements (the shortest element is the n_th_ element) with a spacing factor σ and a scaling factor τ, which can be described by^27,29,33,37^:

1 $$ \sigma = \frac{{S_{i} }}{{2L_{i} }} < 1,\begin{array}{*{20}c} {} \\ {} \\ \end{array} \tau = \frac{{S_{i + 1} }}{{S_{i} }} = \frac{{L_{i + 1} }}{{L_{i} }} = \frac{{W_{i + 1} }}{{W_{i} }} < 1,\begin{array}{*{20}c} {} \\ {} \\ \end{array} i = 1,2,3,...,n $$

where *S*_*i*_ is the spacing between the *i*_*th*_ and the (*i* + 1)_*th*_ elements. *L*_*i*_ and *W*_*i*_ are the length and width of the *i*_*th*_ element, respectively. The apex angle of the PLPA, in degrees, can be determined as a function of τ and σ as:

2 $$ \alpha = 2\tan^{ - 1} \left( {\frac{1 - \tau }{{4\sigma }}} \right). $$

**Figure 3:**
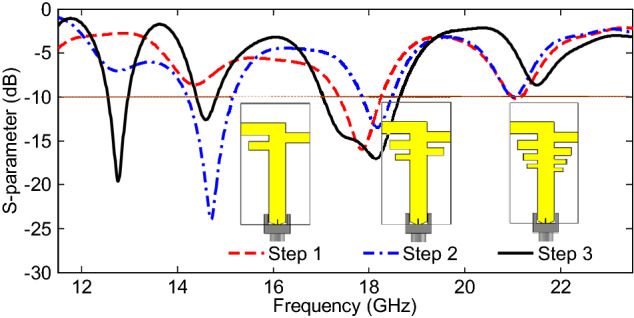
Design steps based on the effects of changing the number of radiating elements.

In order to evaluate the design steps and corresponding |S_11_| parameters, the effects of changing the number of radiating elements in the PLPA, while keeping σ and τ unchanged, are shown in Fig. 3. It is clear that by adding more elements, the operating bands of the antenna are significantly increased, and lower resonant frequencies are obtained, because more elements are in the active region. Effects of other design parameters such as σ, τ, and L_1_ have been investigated based on a parametric study in the following section.

The gain of the PLPA can also be estimated when σ and τ are determined. According to^29^, higher values of σ and τ lead to a relatively higher gain; however, it needs more elements and a larger size of the antenna. The geometry of the final PLPA may be considered as a modified log-periodic array by a trade-off among parameters to obtain better antenna performance in terms of reduced size, multiband operation, and better gain. The scaling factor of the final PLPA antenna τ is chosen as 0.85, with eight elements in total. The initial spacing factor σ is chosen as 0.18, which together with τ will result in a gain of approximately 8 dBi for a typical log-periodic array^29^. In this way, the spacing factor σ is selected based on other criteria. Notably, the effects of coupling among elements impose the smallest value for this variable, because low values of σ cause poor matching. Furthermore, compared to typical log-periodic arrays, the scale factor τ is selected at a low value with higher radiation efficiency and gain^38^.

### Integration of four-unit antenna for MIMO design

**Figure 4:**
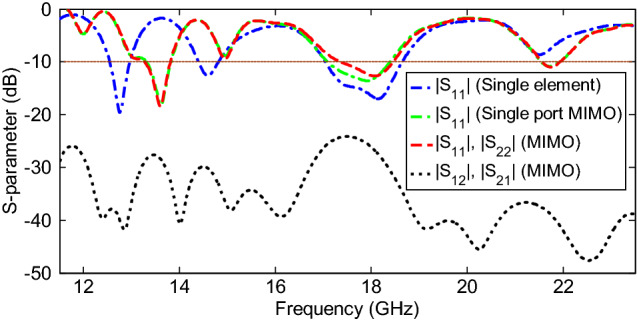
Simulated S-parameter curves for the proposed multiband antenna.

The performance of the single element antenna, as well as the MIMO array, is verified through the transmission and reflection coefficient plots, as shown in Fig. 4. It is clear from the |S_11_| plot that the single element antenna is operating in multiple bands at 12.55–12.98 GHz, 14.39–14.84 GHz, and 17.01–18.65 GHz, ranging from 12 to 19 GHz in the Ku/K bands. Additionally, the |S_11_| and |S_22_| curves of the MIMO elements show that the MIMO array resonates in the same frequency bands, whether with single port or dual ports. A slight band shift is observed between the single element antenna and MIMO configurations, because of the coupling between the two or four elements in the MIMO system, especially at the lower band. Additionally, the transmission coefficient curves in Fig. 4 display that minimum isolation of 24 dB is obtained amid the two ports of the MIMO array for the operating bands. It is worth noting that, despite the small area occupied by the MIMO antenna, the isolation between MIMO ports is relatively high without any decoupling structure. This is due to the location of diagonal elements in the anti-parallel mode for the quad element MIMO array, and the orthogonal arrangement of four radiators relative to each other. Moreover, the combined effect of pattern diversity with two orthogonal elements and a common feed-line not only enhances isolation, but improves far-field radiation characteristics also, with the most minor effect on the near-field results, and is used for cognitive radio applications.

**Figure 5:**
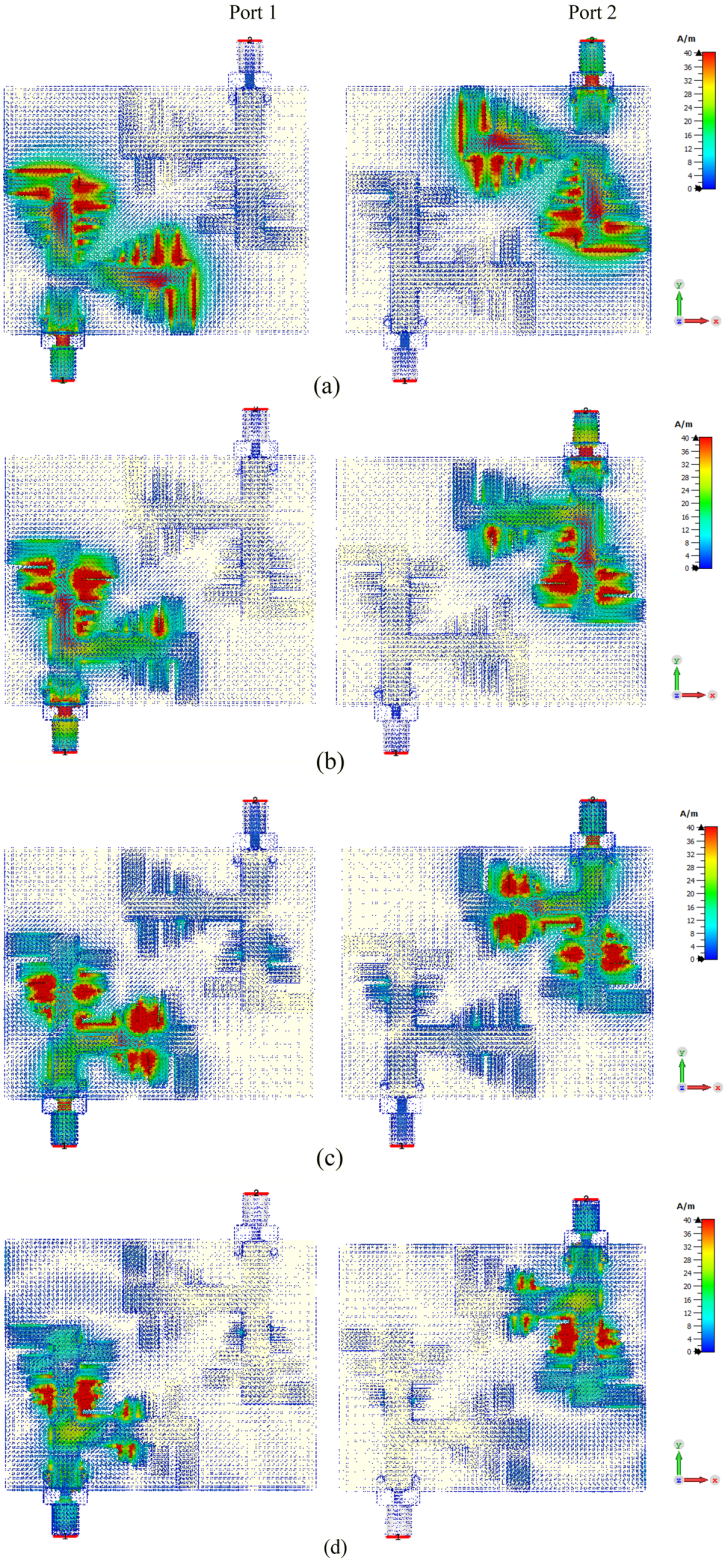
Simulated surface current distributions on the proposed MIMO antenna array for two ports at (**a**) 13.5 GHz; (**b**) 14.8 GHz; (**c**) 18 GHz; (**d**) 21.8 GHz.

To verify the functioning of the designed two-port MIMO array, the simulated distribution of the surface currents is examined at four operating frequencies in each port successively, as shown in Fig. 5. This analysis determines the radiating antenna parts and exhibits the mutual coupling between the adjacent antenna elements. According to current distributions, when the frequency is minimal (12 GHz), most of the radiation is concentrated around the entire structure, but mainly in the higher PLPA zone, while as the frequency increases, the current moves to the shortest PLPA zones. In other words, the active region of the array moves toward the parts of the arrays with a shorter size when the operating frequency increases. It can also be noticed that there is a significant current on the adjacent radiator at approximately 13.5 GHz and 18 GHz, thereby creating two resonances near each other, as shown in Fig. 4. However, it can be seen from Fig. 5 that the coupling current was insignificant among the two ports due to their arrangement.

**Figure 6:**
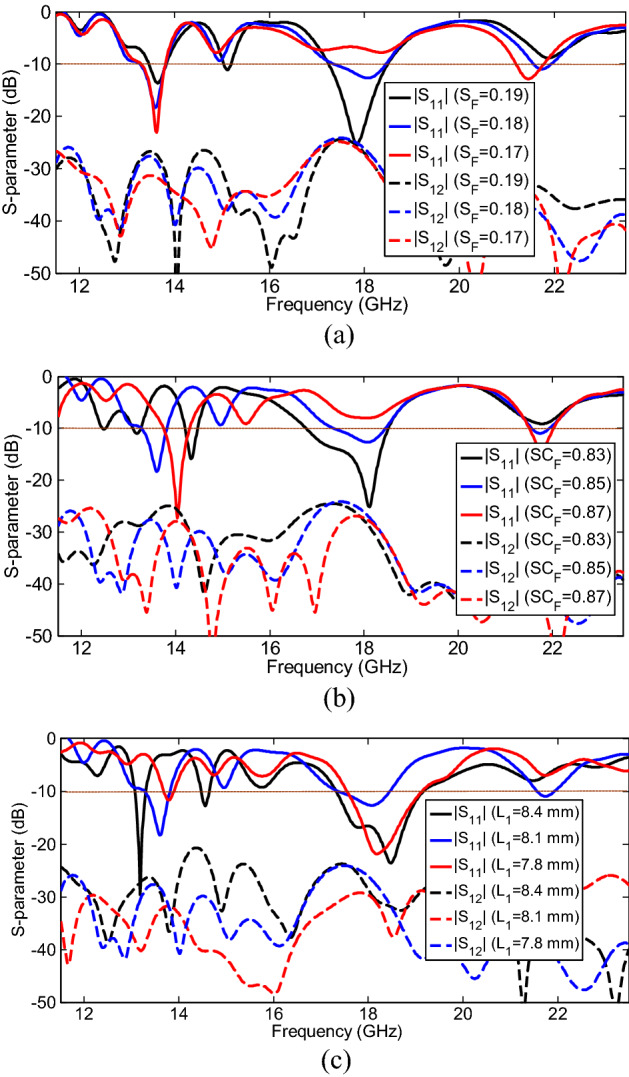
S-parameter characteristics for different values of (**a**) spacing factor (S_F_), (**b**) scaling factor (Sc_F_), and (**c**) L_1_.

The effectiveness of the MIMO array parameters on the S-parameters can be investigated by a parametric study to achieve reasonable matching and acceptable isolation performance simultaneously in the entire frequency bandwidth of operation. In such a MIMO PLPA structure, the inter-element spacing among the array elements and the interleaving location among the log-periodic arrays played a significant role in the MIMO antenna performance. The results in Fig. 6a show that by tuning the spacing factor (S_F_) σ among adjacent elements in the MIMO structure, if S_F_ to be 0.18, admissible impedance matching with high isolation of ports better than 24 dB can be obtained without using any decoupling structure in the operating bands. Figure 6b shows that changing the scaling factor (SC_F_) τ from 0.83 to 0.87 mm has the most effect on the shift of the first resonance frequency band, while only leading to the poor or improving impedance matching on the middle and last bands and has a slight effect on the transmission coefficients. Moreover, as shown in Fig. 6c, the different lengths of L_1_ (the length of the largest element) have considerable effects on the favorable responses of the |S_11_| and |S_12_| parameters at the operating frequencies. It has been observed that for the considered value of 8.1 mm, the best results are seen.

## Results and discussion

**Figure 7:**
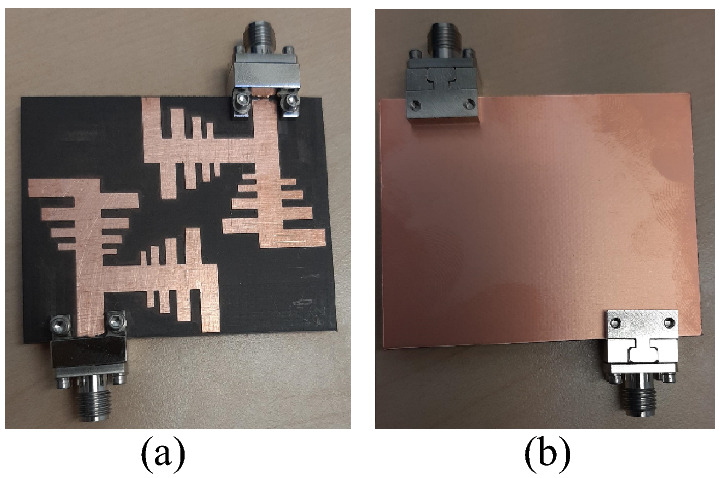
Prototype of the multiband MIMO antenna: (**a**) top layer and (**b**) bottom layer.

To verify the results achieved in the simulation, the proposed multiband MIMO PLPA design is fabricated, and experimental tests are conducted for measurements. Figure 7 shows a prototype of the fabricated multiband MIMO antenna. Some of the typical results of the antenna performance are presented in the following subsections.

### S-parameters

**Figure 8:**
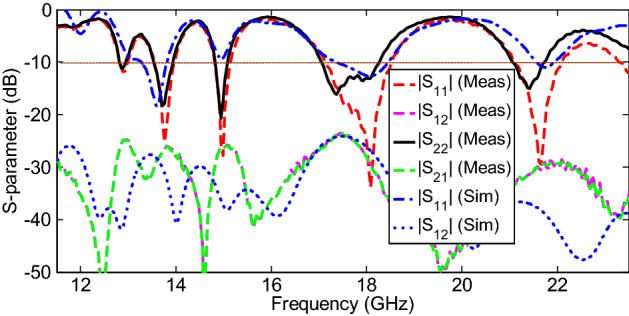
Measured and simulated S-parameter characteristics of the proposed multiband MIMO antenna.

Figure 8 shows the measured and simulated S-parameter characteristics of the proposed multiband MIMO antenna configuration. It is observed that the antenna is well matched over the operating frequency bands, and the difference between the simulated and measured results is probably due to fabrication imperfections and tolerance due to the permittivity of the substrate and port/cable coupling losses. Based on the measured results, the 10-dB impedance bandwidths (|S_11_|) for multiband performance are 1.3% (12.82–12.98 GHz), 3.1% (13.54–13.96 GHz), 2.3% (14.81–15.15 GHz), 4.5% (17.7–18.52 GHz), and 4.6% (21.1–22.1 GHz), which are appropriate for Ku/K band applications. The decrease in the reflection coefficient and difference between |S_11_| and |S_22_| in the measured bands is due to the above reason. Additionally, the measured transmission coefficients (isolation characteristics) are higher than 23.5 dB over the operating bandwidths, which are acceptable for many wireless communication applications.

### Far-field radiations

**Figure 9:**
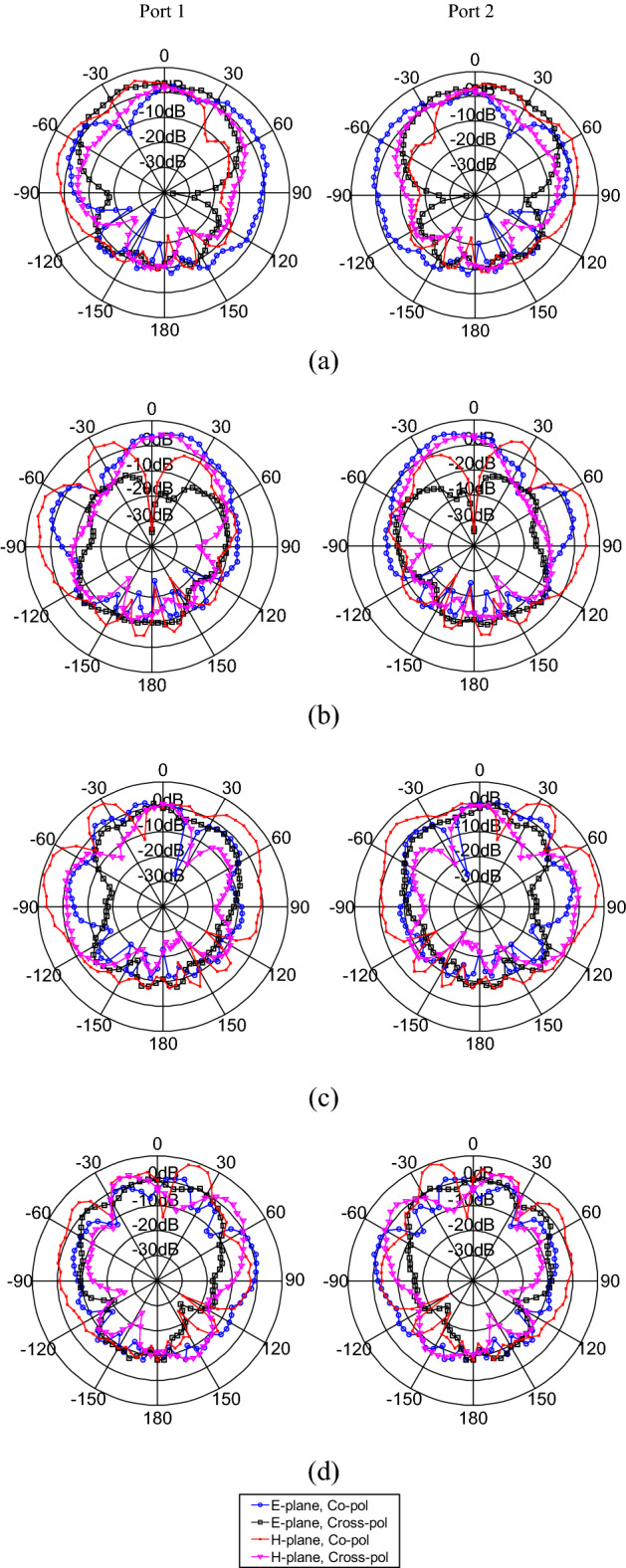
Measured radiation patterns of the fabricated MIMO antenna at (**a**) 13.7, (**b**) 14.95, (**c**) 17.9, and (**d**) 21.4 GHz.

Figure 9 shows the measured 2-D radiation patterns (gain) of the fabricated MIMO antenna in the E and H planes at some operating frequencies, such as 13.7, 14.95, 17.9, and 21.4 GHz, for instance. The radiation patterns are shown for port-1 and port-2, which revealed that different radiation patterns in different directions with diversity performance could be obtained for the proposed MIMO configuration due to the orthogonal arrangement of two MIMO ports. It is worth mentioning that a 50 Ω matched load was used to terminate the unused port while taking measurements.

**Figure 10:**
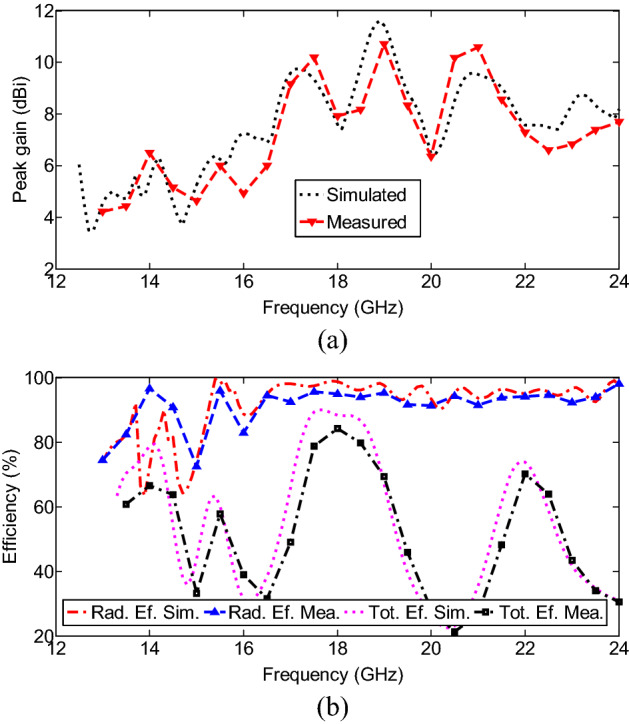
(**a**) Peak gain and (**b**) efficiency of the proposed multiband antenna system.

The simulated and measured peak gain and efficiency performance achieved by the multiband MIMO antenna when Port 1 is excited, and Port 2 is terminated with a 50-Ω load, are shown in Fig. 10. It can be seen that the radiation efficiency of the multiband antenna ranges between 72 and 97%, and the peak gains in the broadside direction range from 4.2 to 10.7 dBi over the operating bandwidths. Correspondingly, the antenna exhibits total efficiency of above 20% (even out of operating band) with a maximum value of 85% at around 18 GHz. As the frequency increases, the antenna element effective aperture increases, and therefore, its peak gain increases.

### MIMO performance metrics

To evaluate the diversity and MIMO applications of the proposed system, key performance parameters, such as the envelope correlation coefficient (ECC), the actual diversity gain (DG), the mean effective gain (MEG), and channel capacity loss (CCL), are analyzed in this subsection.

The ECC is used to describe how much the communication channels are correlated or isolated with each other and indicates the diversity performance and independenc pattern of the antenna elements. It can be computed using the S-parameters (Eq. 3) and far-field radiation characteristics (Eq. 4) of the MIMO antenna in which an amount of less than 0.5 is acceptable to achieve a suitable performance of diversity capability for a multiple antenna system. In this paper, measured and simulated ECCs are calculated using two equations. A lower value of ECC shows a low mutual coupling as well as high channel capacities and vice versa. The ECCs are determined by the following equations, which have already been defined in^4,17^:

3 $$ ECC = \frac{{\left| {S_{11}^{*} S_{12} + S_{21}^{*} S_{22} } \right|^{2} }}{{\left( {1 - \left( {\left| {S_{11}^{{}} } \right|^{2} + \left| {S_{21}^{{}} } \right|^{2} } \right)} \right)\left( {1 - \left( {\left| {S_{22}^{{}} } \right|^{2} + \left| {S_{12}^{{}} } \right|^{2} } \right)} \right)}},{\text{and}} $$

4 $$ ECC = \frac{{\left| {\iint_{4\pi } {\left[ {\vec{F}_{1} (\theta ,\varphi ) \cdot \vec{F}_{2} (\theta ,\varphi )} \right]d\Omega }} \right|^{2} }}{{\iint_{4\pi } {\left| {\vec{F}_{1} (\theta ,\varphi )} \right|^{2} }d\Omega \iint_{4\pi } {\left| {\vec{F}_{2} (\theta ,\varphi )} \right|^{2} }d\Omega }}, $$

where $\vec{F}_{i} (\theta ,\varphi )$ is the complex far-field radiation patterns of the antenna system when the i_th_ port is excited, “·” is regarded as Hermitian product, and Ω is the solid angle. This is a complicated expression that requires 3D radiation pattern measurements and numerical integrations, and it is valid when a uniform multipath environment of balanced polarization is considered.

**Figure 11:**
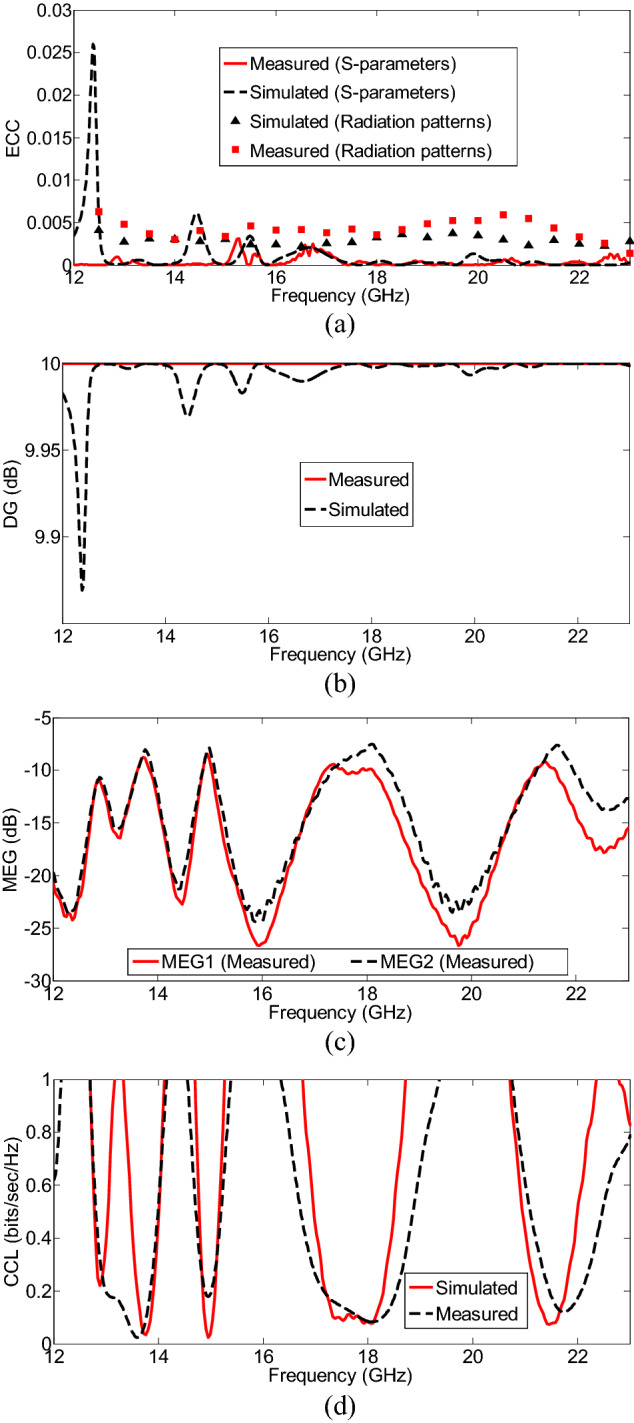
(**a**) ECC; (**b**) DG; (**c**) MEGs; (**d**) CCL, for the proposed multiband MIMO antenna.

Figure 11a shows the ECC characteristics of the proposed MIMO antenna, that computed using the simulated and measured radiation patterns. Although, the simulated values are slightly different from the measured values, the EEC values of the two antenna elements are always below 0.03 over the frequency band. In terms of diversity and channel operation, it makes us to expect good performance. The slight difference between the simulated and measured values of ECCs using Eq. (4) shows the system stability, good far-field independence, and pattern diversity performance of the proposed MIMO antenna.

The DG corresponds to the calculated ECC value of the MIMO antenna system, as given in equation^11^:

5 $$ DG = 10\sqrt {1 - \left| {ECC} \right|^{2} } . $$

Figure 11b shows the DG achieved for the MIMO antenna, which illustrates the reduced amount in transmission power after using a diversity scheme without a performance loss for the MIMO systems^27^. Moreover, it defines the amount of improvement gained by using a multiple antenna system compared to a single antenna system. The values are nearly 10 dB (ideal value) across the operating frequency bands due to the low ECC.

The MEG is basically identified to determine the ability of the antenna to receive the mean power in the multipath and fading environments. In other words, it is supportive to determine the power imbalance by considering the basic parameters such as gain, total efficiency, and propagation environment in multiple antenna elements, degrading the diversity performance. The MEG can be obtained using the equation provided below^39^:

6 $$ MEG_{i} = 0.5\left( {1 - \sum\limits_{j = 1}^{k} {\left| {S_{ij} } \right|} } \right). $$

The terms k and i represent the number of antennas and antenna under observation, respectively. To achieve the imbalanced levels of the diverse propagation branches, the difference between MEGs of any two antennas should be − 3 ≥ *MEG* (*dB*) over the entire band, which is therefore validated for the obtained MEG values (less than − 7.48 dB) in the proposed two-port MIMO antenna, as shown in Fig. 11c.

The CCL is another important diversity parameter that defines the limit of a maximum rate of transmission over a channel. It can be calculated using the following equations^17^:

7 $$ CCL = - \log_{2} \det \left[ {\begin{array}{*{20}c} {\alpha_{11} } & {\alpha_{12} } \\ {\alpha_{21} } & {\alpha_{22} } \\ \end{array} } \right] $$

8 $$ \alpha_{ii} = 1 - \left| {\mathop \sum \limits_{n = 1}^{n = 2} S_{in}^{ * } S_{ni} } \right|\quad {\text{ and }}\;\alpha_{ij} = - \left| {\mathop \sum \limits_{n = 1}^{n = 2} S_{in}^{ * } S_{nj} } \right| $$

The measured and simulated CCLs in the proposed MIMO antenna are plotted in Fig. 11d. The acceptable limit of CCL is 0.4 bits/sec/Hz, and for the proposed MIMO system is less than 0.34 bits/sec/Hz in the operating bandwidth, which shows the high throughput of the proposed MIMO antenna.

## Performance comparison

To validate the suitability and novelty of the proposed multiband offset MIMO antenna, it is compared with the existing state-of-the-art antennas, as shown in Table 2.

**Table 2:** Performance comparison of the proposed multiband offset MIMO antenna with existing state-of-the-art antennas.

References	Antenna elements, ports	Operating frequency (GHz) (|S_11_|< − 10 dB)	MIMO antenna volume (mm)^3^	Minimum isolation (dB)	Maximum ECC	Peak gain (dBi)	Radiation efficiency (%)
^3^	2, 2	7.1–7.3, 8.4–8.6	42 × 17 × 1.6	13	0.015	1.6–5.3	72–96
^4^	6, 6	0.727–1.066, 5.28–6.2, 1.7–1.9, 5.5–5.8, 7.2–8.9	117 × 65 × 0.762	14.5	0.027	− 1.59–9	84–94
^5^	2, 2	1.881–1.943, 2.371–2.51, 3–11	57.25 × 33 × 1.6	14.2	0.047	0.75–5.5	72–94
^6^	10, 10	3.4–3.8, 5.15–5.92	150 × 80 × 0.8	11	0.15	− 1–3.5	40–80
^7^	2, 2	2.71–2.82, 3.73–4.64, 5.14–6.72, 7.52–8.53, 8.66–9.41	36 × 20 × 1.4	15	0.003	1–2.8	88–96
^8^	2, 2	1.86–3.84	100 × 55 × 1.6	13	0.14	3.2–3.87	45–76
^9^	4, 4	1.41–1.62, 2.4–2.462, 3.1–12.8	56 × 56 × 1.6	12	0.4	1.3–2.14	50–87
This work	4, 2	12.82–12.98, 13.54–13.96, 14.81–15.15, 17.7–18.52, 21.1–22.1	55 × 45 × 1.57	23.5	0.03	4.2–10.7	72–97

From Table 2, it can be concluded that the proposed structure supports multiple bands compared to^3,5,6^, has the highest isolation, peak gain, and efficiency compared to^3–7^. Although the dimensions of the proposed antenna are larger than those of some cited works, its structure is less complex to implement in practice; therefore, it is more cost-effective, and has four antenna elements. These characteristics make the antenna feasible for wireless applications in Ku/K-bands.

## Conclusion

In this article, a compact quad-element, two-port multiband PLPA-MIMO antenna system with pattern diversity is demonstrated for Ku/K-band applications. The structure of the presented antenna, S-parameters, parametric studies, radiation patterns, and the effect of the MIMO configurations are thoroughly discussed using a simulated design and manufactured prototype. According to simulated and measured results, the design exhibited an isolation of more than 23.5 dB at the operating frequency bands, without any decoupling structure, while it has a compact structure. Additionally, according to the principle of the log-periodic array antenna, the proposed design has a high peak gain of 4.2–10.7 dBi with a good radiation efficiency of 72–97% over the operating frequency range. In addition, an ECC less than 0.03 and other MIMO performance metrics showed that the proposed antenna is suitable for diversity performance. Therefore, the above characteristics validate that the proposed antenna is a good candidate for multiband MIMO applications covering 12–23 GHz in Ku/K-bands.
